# Connecting the Dots: Livestock Animals as Missing Links in the Chain of Microplastic Contamination and Human Health

**DOI:** 10.3390/ani14020350

**Published:** 2024-01-22

**Authors:** Francesca Corte Pause, Susy Urli, Martina Crociati, Giuseppe Stradaioli, Anja Baufeld

**Affiliations:** 1 Department of Agricultural, Food, Environmental and Animal Sciences, University of Udine, Via Delle Scienze 206, 33100 Udine, Italy; francesca.cortepause@uniud.it (F.C.P.); susy.urli@uniud.it (S.U.); 2 Department of Veterinary Medicine, University of Perugia, Via S. Costanzo 4, 06126 Perugia, Italy; martina.crociati@unipg.it; 3 Centre for Perinatal and Reproductive Medicine, University of Perugia, 06129 Perugia, Italy; 4 Research Institute for Farm Animal Biology (FBN), Wilhelm-Stahl-Allee 2, 18196 Dummerstorf, Germany

**Keywords:** plastic particles, livestock animals, method standardization, One Health, ruminants, food chain, antibiotic resistance genes, heavy metals

## Abstract

**Simple Summary:**

The environmental issue posed by the distribution of plastic is largely due to their fragmentation into micro- and nanoplastics (MNPs), which are spread across aquatic, atmospheric, and terrestrial ecosystems. The discovery that these particles can easily accumulate in aquatic organisms and edible plants has rapidly drawn global attention to their potential impact on human health. Therefore, based on the One Health approach, this paper will first provide an overview of soil contamination as well as human exposure routes and toxicity pathways of MNPs. It then describes the significant role of livestock animals as a critical link between soil and human exposure, focusing on the lack of the available literature and on the role of livestock animals as reservoirs and carriers of MNP contamination along the food chain. The lack of a standardized method to detect, quantify, and characterize plastic particles in different matrices prevents the determination of their realistic load. For this reason, the development of a database where researchers can document data, both on MNP characteristics and assessment procedures, is also suggested with the perspective of training, in the future, artificial intelligence (AI) tools capable of predicting the most abundant/dangerous polymer(s) and help in guiding decisions to reduce risks to global health.

**Abstract:**

Plastic pollution is a global diffuse threat, especially considering its fragmentation into microplastics (MPs) and nanoplastics (NPs). Since the contamination of the aquatic environment is already well studied, most studies have now focused on the soil. Moreover, the number of studies on the exposure routes and toxic effects of MNPs in humans is continuously increasing. Although MNPs can cause inflammation, cytotoxicity, genotoxicity and immune toxicity in livestock animals, which can accumulate ingested/inhaled plastic particles and transfer them to humans through the food chain, research on this topic is still lacking. In considering farm animals as the missing link between soil/plant contamination and human health effects, this paper aims to describe their importance as carriers and vectors of MNP contamination. As research on this topic is in its early stages, there is no standard method to quantify the amount and the characteristics of MNPs in different matrices. Therefore, the creation of a common database where researchers can report data on MNP characteristics and quantification methods could be helpful for both method standardization and the future training of an AI tool for predicting the most abundant/dangerous polymer(s), thus supporting policy decisions to reduce plastic pollution and perfectly fitting with One Health principles.

## 1. Introduction

Plastic has become an integral part of our daily lives since its invention. They are composed of polymers with different lengths like polyethylene (PE), polypropylene (PP), polystyrene (PS), polyethylene terephthalate (PET), and polyvinyl chloride (PVC) as well as various additives including plasticizers, flame retardants, stabilizers, and colorants. These additives enhance properties such as hardness, resistance, and elasticity, making plastics widely used in various sectors [1]. Plastic particles are classified into five categories based on their size: nanoplastics (>100 nm), microplastics (between 100 nm and 1 mm), mesoplastics (between 1 mm and 2.5 cm), macroplastics (between 2.5 cm and 1 m), and megaplastics (>1 m) [2].

However, in recent years there has been growing concern about the widespread presence of micro- and nanoplastics (MNPs) in the environment, which pose potential ecological risks due to their ubiquitous presence and persistence in freshwater, seawater, sediment, soil, and air [3,4]. The most abundant microplastic form in water and sediment are fibers (48.5%), followed by fragments (31%), beads (6.5%), films (5.5%), and foams (3.5%) [5]. Fibrous microplastics (FMPs) can be of natural origin or manufactured and they are widely used in textile production and industry due to their high fineness (micrometric), malleability, and flexibility [6]. The main sources of FMPs are industrial discharges, laundries, dyeing processes, and domestic washing; therefore, they can be found in wastewater, but their presence is also common in soil and atmosphere. The presence of these contaminants is mainly of anthropogenic origin and the coronavirus pandemic has contributed significantly to increase the release of FMPs through the wide use of masks. Although data show a slight decrease in European plastics production (from 60.8 Mt to 58.7 Mt in 2022 and 2023, respectively), their global production continues to increase, reaching 400.3 Mt in 2023 [7]. Therefore, proper management of the sources of MNPs is necessary to limit their impact on the environment and to address the global environmental challenge. There are two distinct sources of micro- and nanoplastics. Primary plastics are intentionally produced and pose higher risks for humans because they are, for example, included in products directly applied to the skin [4]. Today, several regulatory actions have been enforced by numerous countries to ban the use of microbeads in personal care products [8,9]. However, secondary MNPs, which result from the degradation of large plastic waste that have undergone physical, chemical, and biological processes, may pose the greatest risk of contaminating our environment, although there are ongoing efforts to reduce plastic pollution. MNPs can be detected in aquatic and terrestrial systems and even in the air: evidence exists that MNPs are able to accumulate in sediments, plants, or marine organisms [10,11]. Consequently, MNPs infiltrate the trophic chain and affect the ecosystem by causing physical harm, reducing the feeding efficiency and inducing potential toxicity. Moreover, MNPs are capable of transporting and concentrating hazardous chemicals that pose additional risks to plants, animals, and humans [1].

In recent years, research on MNPs has focused on aquatic ecosystems and their inhabitants [12]. Bioaccumulation of microplastics has been demonstrated in several species of fish and mollusks [13,14,15]. Besides aquatic organisms, plants and terrestrial animals constitute significant components of the human food web. The effects of MNP exposure on humans are increasingly being investigated, with a focus on potential health risks. In this regard, several studies have highlighted the exposure and accumulation of MNPs in the soil and plants [16,17,18,19]. The current knowledge concerning MPs and NPs and their potential effects on the reproduction of terrestrial animals has recently been summarized [20]. However, although farm animals are crucial to the food chain for human consumption, either directly (e.g., meat production) or indirectly (e.g., milk for food processing, soil fertilization after manure maturation), detailed information on the effects that plastic particles may have on them through their presence, accumulation, and translocation to different organs and tissues is still lacking. In this review, we aim to emphasize the significance of farm animals as the missing link between plant contamination and human health effects. Farm animals, especially ruminants, act as critical nutrient intermediaries by effectively converting indigestible plant fiber and industry by-products into highly nutritious protein. However, the lack of standardized methods for identifying MNPs in animal products makes it impossible to uniformly estimate the true plastic load. This deficiency is caused by several factors, including the challenge of determining which particle (type, dimension, charge, etc.) can more easily overcome anatomical barriers, and the difficulty in distinguishing between the contamination already present in the raw product and that which is introduced during food processing and packaging [21].

However, to have a comprehensive view of the potential effects of MNPs on both humans and the environment, it is essential to consider the exposure and impact on terrestrial animals. In this scenario, developing a centralized database for researchers to document comprehensive data on the MNP assessment procedure, type/s of plastic observed, size, and, of course, the matrix in which they are detected would assist in standardizing the protocol utilized. Since effective detection methods are crucial for managing contamination by MNPs, the information obtained could be utilized in the future to train artificial intelligence (AI) tools. This could facilitate predictions on which polymer/s will be most prevalent/dangerous, thus guiding decisions and policies aimed at reducing plastic pollution, as well as assessing and managing hazards across the food chain and risks to global health.

## 2. Materials and Methods

In this review, information from journal articles, books, and reports were thoroughly collected and studied. The available literature has been searched through the database of PubMed, Web of Science, ScienceDirect, Google Scholar, and Scopus with the following keywords and strings: “microplastics”, “nanoplastics”, “One Health”, “livestock animals”, “ruminants”, “food chain”, “standardization of the method”, “antibiotic resistance genes”, “heavy metals”, and “artificial intelligence”. The last access to the online databases was conducted in December 2023. The information available in the title and abstract of each article was initially examined to determine the relevance to the topic. The final articles were selected based on the following inclusion criteria: (1) The article evaluated the effects of MNP contamination on soil, focusing on agricultural soil and crops. (2) The article extensively described the MNPs’ route of exposure and the pathways of toxicity in humans. Both in vivo and in vitro studies were selected to estimate the human health threats posed by MNPs. (3) The article determined the principal source of MNPs and their exposure pathways for livestock animals. (4) The article described the role of livestock animals as reservoirs and carriers of MNPs contamination along the food chain. (5) The article proposed future perspectives for artificial intelligence (AI) tools capable of recognizing, predicting the abundance, and control microplastic pollution to achieve the goals for a sustainable development.

The search results were then revised, and the information was analyzed, categorized, and presented in sections to effectively address the scope of this review.

## 3. Principal Source of MNPs

### 3.1. Soil Contamination

While the occurrence and toxicity of MNPs in aquatic environments have been widely reviewed, research on their impact in soil is currently ongoing. It is worth noting that soil assimilates even more plastics than the oceans, and 80% of debris in marine environments comes from land [16,17,22]. Beyond illegal garbage dumping, soil contamination by plastic particles is mostly related to the agricultural sector because of the intense use of plastic mulch films and biosolids [10,23]. In addition, since plastic debris has been detected in soils where agricultural techniques are not required (forests, mountains, urban, and industrial soils), atmospheric deposition must also be considered [24].

Mulch films and biosolids are normally used to improve soil productivity and promote agricultural production. However, plastic mulch films are very thin, cannot be completely removed after the crop season, and can undergo additional fragmentation due to chemical, physical, and biological effects. Similarly, biosolids seem to retain a significant amount of MNPs that can overcome the usual series of processes that they normally undergo before being used, and that can accumulate in the soil after continuous use. Therefore, the short-term productivity gains should be compared to the long-term risks for soil health caused by the accumulation of MNPs [19].

The presence of any type of stressor, such as MNPs, may disrupt the finely controlled soil ecosystem. Changes in its physical and chemical characteristics, such as pH, can affect the native microbial communities required for the degradation of organic matter and for cycling nutrients, which are vital for the development of plants [25].

By reproducing the soil ecosystem in laboratory semi-natural conditions, several studies have tested the effects of different polymers. De Souza Machado et al. [26] tested the effects of six different polymers on the soil health and performance of spring onion (*Allium fistulosum*). Regarding soil, the tested MPs altered its physical characteristics and structure, thus impacting water dynamics and microbial activity; at the same time, root length and diameter increased and decreased, respectively. Depending on the type of MP, the ratio between the roots and leaf dry biomass changed. Particularly, PA decreased this ratio, mainly due to the immense increase in dry leaf biomass associated with an almost doubled nitrogen content. As PA consists of amines and carboxylic acids, it is possible for soil to become enriched with nitrogen, resulting in effects similar to those of fertilization.

Similar results were reported by Boots et al. [25], when high density polyethylene microplastic (HDPE-MPs) caused an alteration in the root/sheet ratio of perennial ryegrass (*Lolium perenne*). To overcome and cope with the stress posed by MPs and provide physiological uptake of water and nutrients, the root system has to expand by increasing the root/sheet ratio.

Not only the type of MPs but also their dimensions and interactions with the soil fauna can play a role in determining their impact on the soil ecosystem. In proportion to their size, MPs can physically block the seeds’ pores in different plants, thus leading to a consistent reduction in the germination rate [25,27].

Jiang et al. [28] tested the effects of two different polystyrene fluorescent microplastics (PS-MPs) dimensions (5 µm and 100 nm) concentrated at 10, 50, and 100 mg/L on broad bean (*Vicia faba*). An oxidative damage assay revealed an increase in the activity of superoxide dismutase (SOD) and peroxidase (POD), both size- and dose-dependent. In contrast, 5 µm PS-NPs cannot enter the plant and tend to accumulate on the roots’ surfaces. Nevertheless, nanoscale particles are more prone to enter plant cell barriers.

Giorgetti et al. [29] reported that 50 nm PS-NPs, at concentrations of 0.01, 0.1, and 1 g/L, were observed in the nucleus of onion root cells (*Allium cepa*), with the possibility of inhibiting chromatin functions and structure. The evidence was also confirmed by Jiang et al. [28], where 100 nm PS-NPs caused genotoxicity.

Similarly, Li et al. [30] tested the effects of small (0.2, 0.5, and 1 µm) and large (2 and 5 µm) PS-MPs, concentrated at 50 mg/L, in a microcosm study on maize (*Zea mais*) for 7 days. Although both root and shoot biomass remained unchanged, small PS beads tended to accumulate in the roots. In contrast, 5 µm PS beads were not detected in any part of the root tissue, and 2 µm PS beads accumulated on the cell wall of the xylem. This confirms that particle uptake is size dependent and represent a serious potential threat to cultivated plants used as food for livestock animals and for humans.

Considering soil fauna, most studies have focused on invertebrates, such as collembolans and earthworms, both on direct effects of MNPs on their health and on effects correlated with their potential to act as carriers of plastic pollutants or soil amendments. A further study tested the effects of macro- and micro-sized plastic particles, resulting from two different polymers (low-density polyethylene microplastic—LDPE—and a starch-based biodegradable one), on wheat (*Triticum aestivum*) with and without earthworms (*Lumbricus terrestris*) [31]. The presence of earthworms alleviated the impairments caused by plastic debris on wheat growth. However, at the same time, higher weight loss, lower number of births, and, in general, higher mortality have been registered. Similarly, Zhu et al. [32] discovered an inhibition in the growth and reproduction of the collembolan *Folsomia candida* exposed to MPs, and Boots et al. [25] reported that the ingestion of HDPE-MPs by the common earthworm *Aporrectodea rosea* caused obstruction and abrasion of the digestive tract, thus limiting the absorption of nutrients and, consequently, reducing the growth and survival rates.

Because almost all studies testing the interactive effects of plastic particles on soil, plants, and fauna are conducted under laboratory semi-natural conditions, the obtained results should be critically interpreted. In fact, semi-natural conditions impose a spatial limit. Plant roots, which tend to enlarge to cope with stressors, could compete with earthworms for space and cause their death, which will be overestimated compared with natural conditions.

Therefore, even if laboratory conditions are a necessary starting point, nowadays, attention is shifting towards finding the most suitable method to sample, identify, and quantify MNPs under environmental conditions. Even if a standard method still does not exist, some insights have already been reported. It is suggested that the most suitable methods for plastic particle analysis are pressurized liquid extraction (PLE) and pyrolysis-gas chromatography coupled with mass spectrometry (Pyr-GC/MS) because they offer more robust quantification for both micro- and nanoplastics [18]. However, since these two techniques did not provide any information about the shape, color, number, or single particle size, spectroscopic techniques, such as Raman and FT-IR (Fourier-transform infrared spectroscopy) spectroscopy, should be applied to assess these characteristics [33,34]. Similarly, it is reported that, even if the choice of the analytical method depends on the scope of the research, there is an urgent need to have standard validated methods [35]. In particular, the application of developing in situ techniques (such as (vis-) NIR spectroscopy) could rapidly detect, identify, and quantify MPs but only for large-scale monitoring of soil contamination [36]. Finally, the use of an integrated and, at least, semi-automatic approach, which combines more analytical methods to retrieve information, both about the physical properties (size, shape, and color) and the chemical composition of the plastic fragments, seems to be another promising solution for the future [37]. Table 1 summarizes the commonly used techniques for the identification and quantification of MNPs in soil.

Even if a large-scale monitoring method to quantify MNPs in the soil environment is still not defined, their presence and impact on the soil ecosystem are evident. The discovery of NPs being absorbed by human-edible plants grown on upper ground has gained global attention from researchers, media, and the public regarding their potential effects on human health. This attention currently overshadows the importance of livestock animals, despite them being major contributors to the direct transfer of contamination along the food chain.

### 3.2. Human Health Threat

**Figure 1:**
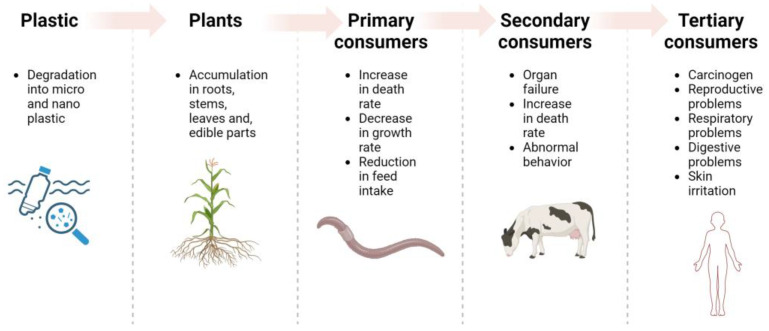
MNPs food chain’s entry routes and effects on organisms.

With the rise of the One Health approach, which encompasses the interdependence among environmental, animal, and human health, numerous studies have examined the presence of MNPs as a ubiquitous contaminant throughout the food chain [34,38]. The aim is to finally understand the multiple routes of entrance and the effects of their presence on humans (Figure 1).

MNPs can enter the human body in three different ways: dermal/skin contact, inhalation, and ingestion. Considering that people mostly live in crowded urban areas and work indoors, the number of plastic particles they take in through inhalation can be very high.

Dris et al. [39] investigated the total atmospheric MP fallout in the urban area of Paris and reported a maximum amount of 280 particles m^2^/day, thus highlighting, for the first time, the role of the air as a source of MNP deposition. Moreover, Vianello et al. [40] used a breathing thermal manikin to sample three different apartments and simulate a person sitting at a table in conditions of standard light activity. The findings indicated that the highest inhalation rate of MPs per hour was 11.3, corresponding to 16.2 MPs inhaled per unit of volume. Then, a male person could inhale 272 MPs over 24 h. To demonstrate that the respiratory system is also a highly stressed route of exposure and that lungs can accumulate MPs, the presence of both polymeric particles and fibers in 13 out of 20 human lung tissues samples obtained after autopsies was confirmed [41]. Polypropylene (PP) and polyethylene terephthalate (PET) were identified as the most abundant polymers. Despite providing crucial insights into MP characterization, the study had limitations. Raman spectroscopy had detection limits preventing the identification of the smallest fragments, which could be present in the lungs and potentially cause biological harm through internalization and translocation. Furthermore, although the detection of MPs in lung tissues is directly associated with inhalation exposure, the presence of particles in other tissues suggests that they may have entered the body through alternative routes. Therefore, the study cannot fully exclude the possibility of systemic translocation of the particles to the lungs, as they were also detected in placental tissue, which can only be reached through systemic circulation.

At the same time, dermal/skin contact needs to be considered because primary MPs, which are intentionally produced, were detected in products that people use daily such as cosmetics, toothpaste, skincare products, and even disposable period products [42,43].

However, ingestion is usually considered to be the primary route of exposure to micro- and nanoplastics. Plastic particles can reach the gastrointestinal tract (GIT) by ingesting contaminated food and water, as well as through ciliary movements of the mucosa of the respiratory tract [44]. Based on the recommendations for food consumption in the US population, Cox et al. [45] estimated the number of MPs ingested and they reported that it was age- and sex-dependent and varied through a range of 39,000 to 52,000 particles ingested annually by female children and male adults, respectively. These estimates increase even further when water consumption is taken into account, with a 22-fold increase in MP consumption observed for individuals who consume only bottled water compared to those who consume only tap water.

Plastic fragments have been found in various parts of the human body. By sampling the stools of eight volunteers coming from different parts of the world, and with different dietary habits, an overview of the global distribution of MP contamination in humans was provided [46]. With a median concentration of 20 pieces per 10 g of stool, all the samples were contaminated with PP and PET, which accounted for more than 80% of the total MP burden.

To prove that MPs can be transported by the bloodstream and cross the placental barrier, Ragusa and colleagues [47] analyzed six human placentas. They found that four of them were contaminated with MPs measuring between 5 and 10 µm. Since 12 fragments were detected in the fetal side (*n* = 5), maternal side (*n* = 4), and chorionamniotic membranes (*n* = 3), the authors demonstrated that the placental tissues can be reached at all levels. This could potentially have harmful effects on the offspring, although the extent of this is still poorly understood. Furthermore, the analysis was limited to only a small part of the placentas. Therefore, it is hypothesized that the number of plastic particles within the entire organ may be much higher.

However, despite the reported presence of plastic particles in the human body, most studies on toxicity pathways rely on assumptions from animal models (like mice and rats) or in vitro cell culture models due to ethical constraints and strict biosecurity measures for handling human samples [42,44].

Using an ex vivo placental perfusion model, Wick et al. [48] found that fluorescently labeled PS-NPs crossed the placental barrier in a size-dependent manner when it was exposed to contaminants during pregnancy. Glucose consumption and lactate production, as well as human chorionic gonadotropin (hCG) and leptin production, remained unaffected, thus indicating that explant viability was not affected by the presence of PS beads.

However, tissue degradation has limited the study to a few hours of perfusion, preventing the evaluation of the effects caused by a chronic treatment with lower doses. Moreover, the perfusion rate measured in the explant only represents the late stage of pregnancy when the barrier between maternal and fetal side decrease and the number of capillaries increases. As a result, the perfusion rate could be higher than during the larger part of gestation. Similarly, the uptake and toxicity of 44 nm PS-NPs in primary human renal cortical epithelial (HRCE) cells, which play a central role in the clearance of drugs in vivo, was tested [49]. Although no adverse effects were reported regarding cell viability and cell cycle progression, the HRCE cells internalized the particles and did not released them even after 90 min, potentially interfering with normal renal clearance functions.

MNPs can cause toxicity through physical, chemical, and biological pathways as well as via translocation to other organs, regardless of their localization inside the body. Physical toxicity results from the biopersistence of plastic particles. This can lead to biological responses like inflammation and oxidative stress, ultimately causing cytotoxicity. Additionally, the persistent nature of MPs makes them challenging to remove, thus leading to chronic inflammation, fibrosis, and an increased risk of neoplasia [50].

Plastic particles can also pose chemical and biological risks. Chemical toxicity occurs due to additives and hydrophobic organic contaminants (HOCs). Additives, like bisphenol A (BPA) and phthalate esters (PAEs), are added during manufacturing processes to prevent degradation and enhance the strength and flexibility of the polymeric material. When released in the environment, HOCs, such as polychlorinated biphenyls (PCBs), dichloro-diphenyl-trichloroethanes (DDTs), and polycyclic aromatic hydrocarbons (PAHs) can bind to the surface of MPs and desorb into organisms. Both additives and HOCs can behave like endocrine disruptors (EDCs), exhibiting hormonal activity that can interfere with reproductive physiology [20,50]. Similarly, because MPs can be colonized by pathogenic microorganisms, like *Vibrio parahaemolyticus*, they also serve as a pathway for biological toxicity [51].

Finally, especially during inflammation, which increases the permeability of the epithelial barriers, through the systemic circulation and lymphatic system, ingested and inhaled MPs can be translocated to secondary organs (like liver, spleen, and kidney) where they can accumulate and cause additional toxicity [42].

Due to the relatively early stage of the research, the assessment of human health risks after exposure to micro- and nanoplastics is still limited. The absence of standardized quantification methods and universal terminology is a major issue.

Even if animal models and in vitro cellular tests are fundamental in identifying the adverse effects posed by MNPs, more observational studies on humans are necessary to understand the toxicity mechanisms and the different interactions of MNPs with other contaminants [44]. Despite these limitations, the extensive detection of plastic particles in humans underscores the need for further studies to determine the sources of this contamination. After discovering that MNPs were present in aquatic organisms and can accumulate in edible plants, their impacts on human health came into global focus. Up to now, only limited attention has been paid to farm animals that may consume contaminated feed, even though they are capable of accumulating MNPs which then enter the food chain and reach humans that are the greatest consumers of livestock products.

## 4. MPs and NPs through the Food Chain: The Role of Ruminants

### 4.1. An Investigation of MPs and NPs in Ruminants

Most of the current research on MP contamination in the terrestrial ecosystem focused on the exposure of soil-dwelling organisms, whereas large ruminants such as cattle and sheep received less attention, although they are potentially the primary consumers exposed to substantial amounts of soil MPs.

As mentioned already, the presence of plastic particles in agricultural soils is due to multiple sources, including the use of plastic mulch and silage films, materials for greenhouses or tunnels, as well as the use of plastic tanks and irrigation pipes. Additionally, other sources include sewage sludge, biosolids, organic fertilizers, and contaminated water that are normally applied during routine agricultural practices [17,52]. Notwithstanding, tire debris from agricultural mechanization, as well as congested roads close to croplands and farms, can also reach and contaminate the soil with MNPs. Subsequently, smaller-sized particles from the soil are easily absorbed by plant roots and translocated to the edible parts through the xylem pathway [30]. Olivieri Conti et al. [53], for example, investigated the MP and NP content of routinely eaten fruits and vegetables purchased from markets in the Catania (Italy) area, and they confirmed that plastics are able to move across individual vegetables. Moreover, they found that apples were the most contaminated samples among fruits, while carrots were the most contaminated among vegetables. However, the raw food used in that study are not representative of the species used as feed for ruminants, thus making the translation of these results to the ruminant food chain difficult.

Despite the increasing evidence of the ubiquitous presence of MNPs on land, there has been limited investigation on their transfer to food-producing animals. It should be noted that they can convert cellulose and low-value plant nutrients into high-value proteins (meat and milk), making them a prevalent source of protein for the population worldwide.

Most of the studies on ingestion of plastic in ruminants come from developing countries. This is mainly due to poor waste management and livestock directly grazing on garbage. In fact, the majority of studies describing the ingestion of macroplastics spread into the environment are conducted in India, Mexico, Ecuador, Jordan, Pakistan, Nigeria, Kenya, Iran, Ghana, and United Arab Emirates, as reported in Table 2. These studies often relate to extreme circumstances, such as undernutrition and mineral deficiencies that facilitate macroplastic ingestion and uncontrolled grazing conditions, such as animals free to feed directly on municipal waste [54]. Therefore, results often report severe cases of rumen obstruction, which cannot be considered representative of the contamination and of the effects posed by the nanosized particles.

However, well-developed countries with established and modern agriculture models are also facing the problem of macroplastic ingestion. In a study conducted by Meyer et al. [55] in Germany, it was reported that 30% of cattle and 6% of sheep displayed plastics in the digestive tract, with the most abundant being net fibers, used for wrapping hay bales, and fragments from plastic sheets used to cover silage.

In a recent study conducted in Spain, soil samples from agricultural land where plastic mulch had been used for at least 10 years were analyzed together with fecal samples from flocks grazing on the same area after crop harvesting [56]. The distribution of MPs in the soil samples followed a normal distribution (2116 ± 1024 particles/kg of soil), whereas in sheep feces the distribution was non-normal (997 ± 971 particles/kg of feces, ranging from 0 to over 5000 particles/kg). MPs were detected in all sampled herds, including the herd that did not directly graze on the mulch-cultivated land. Farmers observed sheep ingesting macroplastic and reported macroplastic contamination in hay and silage. However, the study did not characterize MP particles due to the applied method (observation by microscopy). Therefore, it was not possible to identify mulching as the primary source of plastic ingestion in sheep. Additionally, there was no evaluation made on the milk or meat MPs content in these herds, which means that the information regarding the transfer of MPs across the food chain was incomplete. Nonetheless, this study draws the attention to grazing livestock for future research on plastic epidemiology.

The paucity of reports on MNP contamination in livestock is due to the difficulty in selecting a detection and characterization method and distinguishing between different sources of plastic contamination.

To address this issue, Da Costa et al. [57] evaluated the presence of small-sized particles (≥5 µm) in raw milk collected at the milking parlor and in commercial liquid and powdered milk, which can be contaminated at different stages along the dairy supply chain. Ranging from 204 to 1004 MPs per 100 mL, all the samples were contaminated, and the main detected polymer was PE, with PP, followed by polyester and polytetrafluoroethylene (PTFE). The study showed that the number of plastic particles increased from the farm to the processed milk powders. This provides valuable insights into the accumulation of plastic particles at different stages of food processing. However, the study had limitations. As the raw milk was obtained from the milking machine, it was impossible to determine whether the detected MPs originated from the cow itself or from the milking parlor environment. Similarly, milk samples from national and international brands in Mexico [58] and from markets in Turkey [59] were tested. Both studies confirmed the presence of plastic particles of different colors, shapes (fragments and fibers), and sizes (0.1–5 mm and 0.025–5 mm in [58] and [59], respectively) in all the samples with an average value of 6.5 particles/L.

Although these studies provided a bssasis for further investigations into MPs contamination during milk and dairy product processing, the content of MPs in the branded milk varied significantly between the two studies, possibly because Da Costa Filho et al. [57] detected a range of smaller particles, which were not considered by Kutralam-Muniasamy et al. [58,59].

In addition to the dairy industry, evaluating contamination in the food chain also involves examining meat. Previous studies have primarily focused on aquatic organisms, while contamination in terrestrial livestock has been limited to the presence of MP particles in poultry gizzards [60]. However, there have been no reports on contamination in muscle tissue, which is more commonly consumed [61].

Similar to milk processing, the processing and packaging of meat may increase the accumulation of MNPs, thus making it impossible to identify the actual source of the contamination. Habib et al. [62] found that commercially available meat from the butchers or from the supermarkets in the Middle East was contaminated with polyethylene-based MPs originating from plastic cutting boards. In addition, the study conducted in France by Kedzierski et al. [63] shows that extruded polystyrene microplastics (MP-XPS), used primarily in food trays, are capable of contaminating products fed at a level between 4.0 and 18.7 MP-XPS/kg packed meat (chicken). In both studies, the meat was carefully rinsed, but without eliminating the presence of MPs, and by observing the fate of MPs during cooking or frying, the study highlighted the possible formation and the release of degradation products with unknown effects.

Therefore, the evidence arising from these studies underscores the need to identify the most suitable method for detecting, sampling, and measuring the abundance and properties of MNPs in various livestock products. This will provide data for the risk assessment of MNPs along the food chain, which is currently absent from the literature [64].

**Table 2:** Type(s) of polymer(s) detected in animal samples from different exposure routes and countries.

Ruminant Animal	Country	Findings	Type of Plastic Particles	Source/Route	Technique	References
Sheep	Spain	Feces	PE ^1^	Ingestion of plastic mulch films	Stereomicroscope	[56]
Goat	Middle East	Meat	PE	Cutting boards	FT-IR spectroscopy	[62]
Cattle	Switzerland/France	Milk products	PP ^2^, polyester, PTFE ^3^, PS ^4^	Milking process	Micro-RAMAN	[57]
Cattle	Mexico	Milk	PES ^5^, PSU ^6^	Industrial process	Micro-RAMAN	[58]
Cattle	Ecuador	Milk	PP, HDPE ^7^/LDPE ^8^, PAAm ^9^	Industrial process	Stereomicroscope and FT-IR spectroscopy	[65]
Cattle	South China	Manure	PP, PE	Compost application	Stereomicroscope and FT-IR spectroscopy	[66]
Cattle	North China	Manure	PE, PET ^10^, PP	Long-term compost application	Stereomicroscope and FT-IR spectroscopy	[23]
Cattle	Turkey	Milk	Nylon-6, PET, EVA ^11^, PP, PU ^12^	Industrial process	Stereomicroscope and FT-IR spectroscopy	[59]
Cattle, sheep, goat	Ethiopia	Rumen and reticulum	Plastic bag	Ingestion municipal solid waste by free grazing	Visual inspection	[67,68,69]
Sheep	Jordan	Rumen	Plastic bag	Grazing on polluted land	Visual inspection	[70]
Buffalo, Achai cow	Pakistan	Reticulorumen	Plastic bag	Ingestion of no dietary materials	Visual inspection	[71,72]
Small ruminant and cattle	Nigeria	Rumen	Plastic bag	Ingestion of plastic garbage	Visual inspection	[73,74,75,76]
Sheep and goat	Kenya, Iran	Rumen	Plastic bag	Ingestion of plastic garbage	Visual inspection	[77,78,79]
Buffalo, Axis deer, goat	India	Rumen and reticulum	Plastic waste	Grazing on polluted land	Visual inspection	[80,81,82]
Small ruminants	Ghana	Fore stomach	Plastic for packaging food	Supplementary feed	Visual inspection	[83]
Cattle and sheep	Germany	Gastric tract	Agricultural waste	Ingested anthropogenic debris	Visual inspection	[55]
Dromedary camels	United Arab Emirates	Stomach	Plastic waste, PE, PP, EVA	Ingestion municipal solid waste by free grazing	FT-IR spectroscopy	[84]

^1^ Polyethylene, ^2^ polypropylen, ^3^ polytetrafluoroethylene, ^4^ polystyrene, ^5^ polyethersulfone, ^6^ polysulfone, ^7^ high-density polyethylene, ^8^ low-density polyethylene, ^9^ polyacrylamide, ^10^ polyethylene terephthalate, ^11^ ethylene vinyl acetate, ^12^ polyurethane.

### 4.2. The Role of Plastic Additives as Additional Threats along the Food Chain

The presence and the release of additives used to enhance plastic properties during manufacturing may pose additional risks. Fierens et al. [85] reported that studies investigating the presence of phthalates in dairy products have been published since 1986. However, most studies primarily focused on finished products in the dairy industry (butter, milk creams, etc.). Therefore, it was not possible to differentiate between contamination from packaging or industrial processes and plastics ingested by ruminants through feed. In this view, this issue was addressed by including raw milk samples from different farms in Belgium [85] and in Italy [86] in their studies. In the first study, the results revealed that raw milk at the farm level was contaminated with diisobutyl phthalate (DiBP) and di(2-ethylhexyl) phthalate (DEHP), which was also observed in a previous investigation conducted in Norway [87]. On the other hand, Santonicola et al. [86] confirmed the presence of bisphenol A (BPA) in the milk chain, finding average concentrations of 0.757 µg/L in the samples milked manually, 0.580 µg/L in the samples mechanically milked, and 0.797 µg/L in milk from cooling tanks. These findings suggest that the detection methods should focus on the quantification of DiBP and DEHP in cattle feed to improve the security of the milk chain at the farm level, but detection should also focus on the quantification of BPA introduced potentially during milking from the plastic parts of the milking machine. Unfortunately, no preliminary assessment of phthalates in cattle feed was planned, so that complete journey “from farm to fork” of plastic additives cannot be evaluated. Fierens et al. [85] also highlighted that European legislation [88] progressively reduced the use of DnBP and DEHP, even if different plasticizers have been introduced at the manufacturing level.

However, in view of improving risk management strategies (databases, software, artificial intelligence solutions, etc.), it must be reminded that phthalates are lipophilic, so it is reasonable to assume that high-fat yielding breeds/farms, or even productive season, could represent a risk factor for raw milk and contamination in the milk chain [87,89,90]. 

Cattle are also considered highly sensitive to PCB intake and tissue accumulation [91,92,93]. PCB use has been prohibited in EU, but these compounds persist in the environment for a long time. Since PCB translocation through the food chain has been investigated, EFSA [94,95] has established upper limits for beef meat PCB content together with limits for grazing soils and cattle feed.

In the view of a complete risk assessment, the evaluation of PCB content in soil/feed could be not exhaustive: the amount of soil intake should also be considered. Moreover, silage/grass/hay could contain a higher soil percentage due to inefficiencies in foraging, in low quality meadows, or in the dry or muddy season, which in turn can increase the soil ingestion from 3 to 10% of dry matter intake [91,96].

Farm management should also be considered, as cow–calf operations, where calves are raised under their dams and milk-fed for months before weaning, represent another risk factor for meat contamination due to PCBs’ ability to move from a dam’s fat tissue to the milk [91]. As a final note, our knowledge on the “epidemiology” of compounds with chemical structure close to PBCs, which are referred to as “dioxin-like PCBs” (dl-PCBs) should be implemented. It has been reported that even in beef herds where both soil and feed levels of these contaminants were below limits, meat has been found exceeding the EU limits [91]. In the case of the dl-PCB family, ruminants other than cattle have been considered: sheep [97] and deer [91,98] liver were found to frequently exceed the EU upper limits. However, no “from farm to fork” approach was implemented in the studies reported and the source of contamination was not identifiable.

Therefore, more studies are needed to address the impact of plastic additives as a source of contamination along the food chain.

### 4.3. Microplastics can Carry Antibiotic Resistance Genes (ARGs) and Heavy Metals (HM) along the Food Chain

One Health is a transdisciplinary approach that focuses on optimizing the complementary health of people, animals, and the environment [99,100]. Nowadays, this concept is extremely important for a comprehensive study of contaminants and their interconnections. Together with the MPs crisis, antibiotic resistance (AR), caused by the large and inappropriate use of antibiotics both in human clinics and in the veterinary/agricultural sector, is one of the most relevant challenges for the environment in the 21st century, which becomes even worse when these two phenomena interact [101].

MPs have a high specific surface area, which promotes the accumulation of pollutants, such as heavy metals (HM) and antibiotics, and can be colonized by different microbial communities, collectively known as “the Plastisphere”, including antibiotic resistance gene hosts, such as *Flavobacteriacea* [102,103,104].

Receiving runoff from agricultural activities, industries, and other anthropogenic inputs, the aquatic ecosystem is particularly affected by MPs and antibiotics/ARGs.

As well, the terrestrial environment is also affected, thus leading to the transfer of these combined contaminants along the entire food chain and finally posing serious concerns for human health.

MPs can form aggregates in the soil and interact with its minerals, thus exacerbating the vertical and horizontal spread of the particles and their connected pollutants (like ARGs) through the agroecosystem [105,106]. Considering agricultural practices, the application of animal manure, biosolids, and irrigation with reclaimed wastewater facilitate the dissemination and migration of MPs and ARGs in the soil. This can also negatively impact invertebrates as reported by Ma et al. [107], when MPs combined with tetracycline facilitated antibiotic resistance in the gut of *Enchytraeus crypticus*.

MNPs and ARGs can be absorbed by roots from soil and transported to the edible upper ground parts of the plant, which can then be consumed by farm animals, in particular ruminants [108]. Therefore, the synergic activity between plastic debris and the accumulation of pollutants attached to their surface implements the potential risk of transmission for ARGs that, once inside the animals, may be transmitted to humans following two different pathways: direct contact or, indirectly, through the animal–environment–human route [99].

The use of antibiotics as growth promoters has been banned in Europe and their use in general has been gradually reduced. However, the constant circulation of bacteria species in the agroecosystem, caused by the application of different agricultural techniques (such as manure application on farmlands), has drawn attention on the role of livestock as a reservoir of ARGs. Different studies focus on determining the contribution to their global dissemination. In testing their amount in bovine, swine, and poultry manure and compost, Qian et al. [109] detected a total of 109 ARGs with diversity and abundance that were significantly higher in poultry and swine manure than in bovine manure. At the same time, industrial composting was more efficient in reducing the ARGs in poultry manure. In addition, other authors investigated and reported the presence of some specific antibiotic-resistance genes in various animals’ manures [110,111].

Since the combination of MPs and antibiotics is considered more harmful in increasing the abundance of ARGs, Liu et al. [112] tested both the single and combined effects of MPs and chlortetracycline (CTC), discovering that their interaction increased the abundance of *Prevotella* and *Faecalibacterium* and the incidence of tetracycline ARGs subtypes in the gut microbiome of Muscovy ducks.

Finally, to demonstrate that humans can also drive antibiotic resistance, which, in turn, can negatively impact animals, Dulo et al. [113] sampled goat carcasses, feces, equipment, and the environment in a large abattoir in a pastoralist region of Ethiopia. They reported the presence of antibiotic-resistant *E. coli* O157:H7 in cecal contents, carcass swabs, and water and, although the prevalence was very low (from 2.5% to 7.1%), all isolates were resistant to two or more antimicrobials. In particular, the study identified *E. coli* strains resistant to drugs that are not used in goats, thus suggesting that the source of the resistance was related to human infections and was not coming from animals. As stated in previous paragraphs, developing countries, including Ethiopia, suffer from heavy environmental macroplastic pollution and ruminants easily feed directly on plastic; this phenomenon, together with improper antibiotic use, could lead to increased, and spreading, antimicrobial resistance.

Even if the interaction between MNPs and other pollutants is still less explored, a certain accumulation has also been demonstrated for heavy metals (HM). In this regard, Abbasi et al. [114] reported that lead (Pb), cadmium (Cd), and zinc (Zn) were absorbed on the surface of plastic particles, transported by them, and desorbed in the rhizosphere zone of wheat (*Triticum aestivum*). This raises concerns about the possibility of these pollutants entering the food chain.

The ruminant digestive process involves repeated fermentations of the ingested raw fiber and prolonged retention of chyme to improve digestibility. However, this physiological process can also release pollutants (such as HM), as demonstrated by Liao and Yang [115] who showed that MPs released cadmium and mercury (Hg) during in vitro ruminal digestion.

Additionally, Mahadappa et al. [80] observed that buffaloes, which consumed plastic waste, experienced a significant decrease in ruminal motility and protozoan density. Moreover, their rumen pH increased, and they showed a tendency to accumulate mercury, lead, cadmium, and chromium (Cr) in their kidneys, whereas copper (Cu) primarily accumulated in their livers. Similarly, the accumulation of Cd in the liver and the kidney, along with the increased accumulation of Hg through the muscle, liver, and the kidney, has been observed in sheep [116].

After accumulating in the body, HMs can cause various health problems, including damage to the nervous, cardiovascular, renal, and reproductive systems, especially at higher doses [117]. However, despite extensive information on the toxic effects of heavy metals on mammals, there are currently insufficient data to develop and/or validate a food chain model describing the transfer of these substances to animal products intended for human consumption.

Therefore, studies are still needed to understand the accumulation, transport, and transfer pathways of pollutants attached to the surface of MPs, as their ability to disseminate contaminants affects global health. Furthermore, since livestock systems seem to contribute to the global dissemination of ARGs and HMs through diverse transmission routes, future studies should be conducted following a more holistic approach to the One Health principles.

## 5. Conclusions and Future Perspectives

In conclusion, the current research landscape on MP contamination in terrestrial ecosystems has predominantly concentrated on soil-dwelling organisms. However, large ruminants like cattle and sheep, which are primary consumers exposed to significant amounts of soil MPs, have received comparatively less attention. The transfer of MNPs to food-producing animals is an area that has not been extensively studied, particularly in the context of livestock’s pivotal role in converting plant nutrients into essential proteins for human consumption. The lack of reports on MNPs in livestock is due to the methodological challenges in detection and characterization. The available evidence emphasizes the urgency of identifying reliable methods for detecting, sampling, and measuring MNPs in various livestock products. An approach like this is crucial for conducting comprehensive risk assessments throughout the entire food chain.

Alternative materials to oil-derived plastics are constantly investigated worldwide to reduce global pollution. Saad et al. [118], for example, described a method to produce biodegradable and bio-based polyhydroxyalkanoates (PHAs) as alternatives to conventional plastics by reusing animal and feed by-products, which are typically burned or used as fertilizers. However, the implementation at the industrial level is not competitive from an economic point of view at present.

Considering the issue of microplastics from a broader perspective, it is conceivable that machine learning could assist in evaluating the core of the problem. There are numerous potential approaches for incorporating artificial intelligence (AI) into this multifaceted topic. In terms of detection and monitoring, AI can assist with image recognition and analysis of the additional sensor data. Others have demonstrated the implementation of machine learning to detect and monitor littering in aquatic environments [119,120,121,122]. Additionally, AI can aid in environmental impact or risk assessment if appropriate predictive models are available. This concept can be expanded by utilizing AI to determine the origin of various plastic pollution by analyzing data related to the size, shape, and composition of microplastics. The collected information can inform policy decisions and help target pollution prevention efforts. Thus, it can assist regulatory agencies in creating effective policies. Furthermore, AI-based compliance monitoring is feasible through the above-mentioned applications, which may result in the refinement or adaptation of individual regulations.

To create reliable models and algorithms, the underlying database is mandatory. To date, a comprehensive and uniformly distributed database to facilitate this goal does not exist. Therefore, further studies should establish standardized protocols to acquire comparable data to feed the aforementioned models/algorithms [123].

As our understanding of MNPs in livestock and their potential implications for human health grows, future research must adopt an integrated and intersecting approach to address this global environmental and public health concern.

## Figures and Tables

**Table 1:** Techniques for identifying and quantifying plastic particles in soil.

Method	Technique	Strength Points	Weak Points
Stereomicroscope	Visual sorting	Ubiquitous presence in laboratories, non-destructive, detection of specific characteristics (color, size, shape, etc.).	Difficulties in identification of particles <100 µm, misidentification of plastic from other materials, not used as single step analysis.
FT-IR ^1^	Spectroscopic technique	Simple, efficient, non-destructive.	Plastic must be free of any coating/film, spectral quality influenced by organic matter/water (extensive sample pre-treatment), limit of detection 20 µm, require more time per measurement.
Raman spectroscopy	Spectroscopic technique	Better spectral resolution and lower interference signals than FT-IR, non-destructive, provide data on plastic characteristics (color, size, shape, etc.).	Detection limit of 1 µm, no information about mass or concentration of plastics in samples.
Pyr-GC/MS ^2^	Thermo-analytical technique	Rapid and simple, requires no pre-treatment, provides quantification of the plastic particles in the environmental samples (mass concentration), analyzes particles in low size ranges.	Destructive, no data on plastic characteristics.
(vis-)NIR spectroscopy	Near-infrared (NIR) process-spectroscopic method combined with chemometrics	Non-destructive, rapid in situ detection, identification, and quantification of MPs on large scale, requires no sample preparation.	Able to assess only whether the soil is contaminated or not, incapable of providing quantitative, morphological, and structural information on MPs, low prediction accuracy and high detection limit.

^1^ Fourier-transform infrared spectroscopy, ^2^ pyrolysis-gas chromatography coupled with mass spectrometry.

## Data Availability

Data are contained within the article.
